# Deadenylation of mRNA by the CCR4–NOT complex in *Drosophila*: molecular and developmental aspects

**DOI:** 10.3389/fgene.2014.00143

**Published:** 2014-05-26

**Authors:** Claudia Temme, Martine Simonelig, Elmar Wahle

**Affiliations:** ^1^Institute of Biochemistry and Biotechnology, Martin Luther University Halle-WittenbergHalle, Germany; ^2^Genetics and Development, Institute of Human Genetics – CNRS UPR1142Montpellier, France

**Keywords:** poly(A) tail, deadenylation, translational control, development, mRNA decay

## Abstract

Controlled shortening of the poly(A) tail of mRNAs is the first step in eukaryotic mRNA decay and can also be used for translational inactivation of mRNAs. The CCR4–NOT complex is the most important among a small number of deadenylases, enzymes catalyzing poly(A) tail shortening. Rates of poly(A) shortening differ between mRNAs as the CCR4–NOT complex is recruited to specific mRNAs by means of either sequence-specific RNA binding proteins or miRNAs. This review summarizes our current knowledge concerning the subunit composition and deadenylation activity of the *Drosophila* CCR4–NOT complex and the mechanisms by which the complex is recruited to particular mRNAs. We discuss genetic data implicating the complex in the regulation of specific mRNAs, in particular in the context of development.

## INTRODUCTION

The poly(A) tails of eukaryotic mRNAs, which are added with a more or less uniform but species-specific length during 3′ end processing in the nucleus, are subject to shortening in the cytoplasm, a process termed deadenylation. Deadenylation is the first step in mRNA decay, and subsequent steps are triggered by shortening of the poly(A) tail below a certain, not very well defined, threshold. The rates of deadenylation vary between different mRNAs and determine, to a large extent, the specific half-lives of mRNAs. Thus, controlled deadenylation contributes to the regulation of the steady-state levels of mRNAs and, as a consequence, protein output. Deadenylation rates are determined by regulatory proteins or RNAs that bind to specific sites in mRNAs, typically in the 3′ UTR, and recruit deadenylases (Goldstrohm and Wickens, 2008; Houseley and Tollervey, 2009; Wahle and Winkler, 2013). Deadenylation, in combination with opposing poly(A) tail extension, is also used to regulate the translation of mRNAs. Whereas this type of regulation has been investigated in detail in oocytes, early animal embryos, and neurons (Barckmann and Simonelig, 2013; Villalba et al., 2011; Weill et al., 2012), it does not appear to operate in other cells (Subtelny et al., 2014).

Three main poly(A)-specific 3′ exonucleases, or deadenylases, are known: the poly(A)-specific ribonuclease (PARN; Harnisch et al., 2012; Godwin et al., 2013; Virtanen et al., 2013), the Pan2/Pan3 complex (Harnisch et al., 2012; Wahle and Winkler, 2013; Wolf and Passmore, 2014), and the CCR4–NOT complex. The CCR4–NOT complex, which has been covered in several recent reviews (Goldstrohm and Wickens, 2008; Collart and Panasenko, 2012; Harnisch et al., 2012; Wahle and Winkler, 2013), is the predominant deadenylase in all biological systems and, to our knowledge, for all mRNAs examined. Here, we will focus specifically on the structure and function of the CCR4–NOT complex in *Drosophila*. We will limit ourselves to a discussion of the role of the complex in mRNA deadenylation, including recruitment of the CCR4–NOT complex by mRNA-specific factors. In addition to deadenylation, the complex can repress translation independently of deadenylation (Cooke et al., 2010; Braun et al., 2011; Chekulaeva et al., 2011; Bawankar et al., 2013; Zekri et al., 2013; Bhandari et al., 2014; Chen et al., 2014a; Mathys et al., 2014), and a role in transcription is also being investigated (Collart and Panasenko, 2012). These other functions will not be covered. We will briefly discuss CCR4–NOT- versus Pan2/Pan3-dependent deadenylation. PARN is not conserved in *Drosophila*.

## SUBUNITS OF THE CCR4–NOT COMPLEX, THEIR GENES, AND FUNCTION IN mRNA DEADENYLATION

**FIGURE 1:**
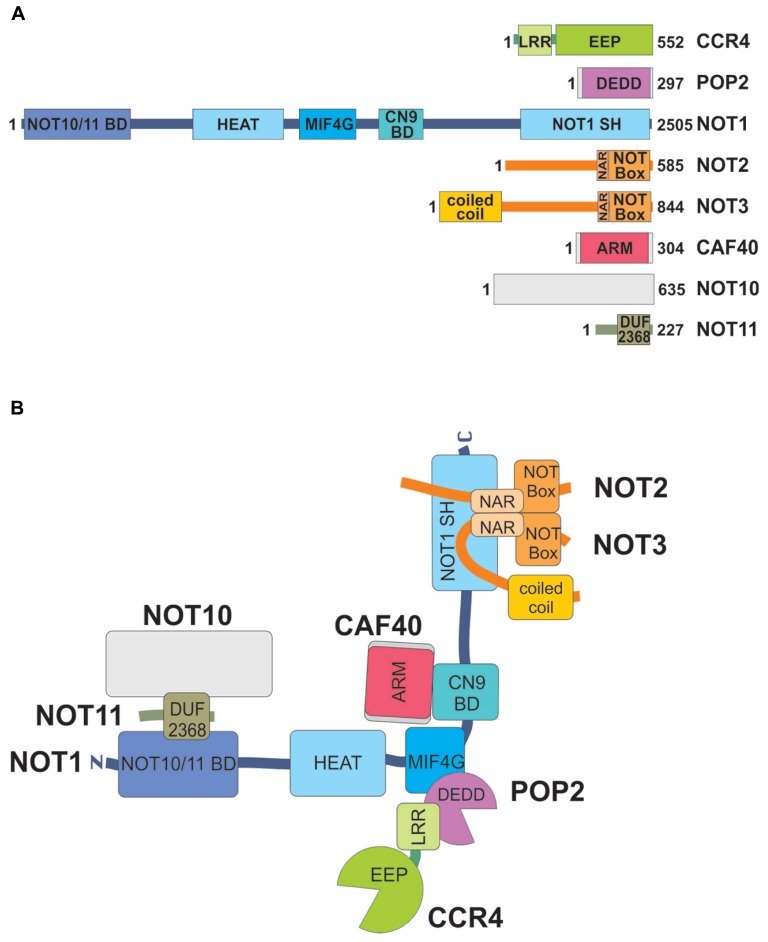
**Subunits of the *Drosophila* CCR4–NOT complex, their domains and interactions. (A)** Domain structures of subunits. The size of each polypeptide (in amino acids) is indicated on the right. Note that this can vary due to alternative splicing. Each large rectangle corresponds to a structured domain. CCR4 contains a leucine-rich repeat (LRR) and an exonuclease-endonuclease-phosphatase (EEP) domain. POP2 consists of a single nuclease domain of the DEDD class. NOT1 contains a NOT10/NOT11 binding domain at its N-terminus, a series of HEAT repeats, a middle-of-4G (MIF4G) domain, and a CAF40/CNOT9 binding domain (CN9BD) in the middle. The C-terminus is formed by a conserved NOT1 superfamily homology (NOT1 SH) domain. The MIF4G, and the NOT1 SH domains are also composed of HEAT repeats. Both NOT2 and NOT3 contain a C-terminal NOT box preceded by a NOT1 anchor region (NAR), NOT3 also has a predicted N-terminal coiled–coil domain. CAF40 consists of armadillo (ARM) repeats. NOT10 has no known or predicted domain, and NOT11 contains a domain of unknown function. **(B)** Interactions between the subunits of the CCR4–NOT complex. Domains are indicated with the same color code as in **(A)**. The orientation of the coiled–coil domain of NOT3 is arbitrary. Interactions shown are based on experimental data for the *Drosophila* complex and comparison to the yeast and mammalian complexes (Bawankar et al., 2013). The L-shape indicated for the complex is based on cryo EM images (Nasertorabi et al., 2011).

**Table 1** lists the eight known subunits of the *Drosophila* CCR4–NOT complex together with their genes, their yeast and human orthologs. Known functional domains of the polypeptides are shown schematically in **Figure 1A**. Note that the subunit POP2 is called CAF1 in most publications. However, the gene name *Pop2* (under which the corresponding yeast gene was first described) is used in Flybase (flybase.org), whereas the abbreviation CAF1 is used for Chromatin Assembly Factor 1. In this article, we will adopt the Flybase nomenclature. Two polypeptides associated with the CCR4–NOT complex in other organisms have not been identified in the *Drosophila* genome. These are CAF130 (Chen et al., 2001), which appears to be yeast-specific, and the mammalian protein TAB182 (Lau et al., 2009). The function of these proteins in the CCR4–NOT complex, when present, has not been analyzed, and TAB182 has not been found consistently in all preparations (Mauxion et al., 2013). NOT4 is a component of the CCR4–NOT complex in *Saccharomyces cerevisiae*. Whereas the protein is conserved, it is not stably associated with the CCR4–NOT complex in flies or mammals (Harnisch et al., 2012; Wahle and Winkler, 2013). Even in yeast, *not4* mutants have at most a marginal deadenylation phenotype (Tucker et al., 2002). In *Drosophila*, the subunits CCR4, POP2, and NOT1-3 are expressed at all developmental stages, including early embryos before the activation of the zygotic genome, and they are found mostly in the cytoplasm, as would be expected for an mRNA deadenylating enzyme (Temme et al., 2004, 2010).

**Table 1:** Subunits of the CCR4–NOT complex in *Drosophila* and their orthologs in yeast and man.

Name of subunit	Annotation symbol; gene	Yeast ortholog(s)	Human ortholog(s)
CCR4	CG31137; *twin*	Ccr4	CCR4a = CNOT6C; CCR4b = CNOT6L
POP2	CG5684; *Pop2*	Caf1 = Pop2	Caf1a = CNOT7 = CAF1; Caf1b = CNOT8 = CALIF = POP2
NOT1	CG34407; *Not1*	Not1	CNOT1
NOT2	CG2161; *Regena* (*Rga*)	Not2	CNOT2
NOT3	CG8426; *Not3*	Not3Not5	CNOT3
CAF40	CG14213; *Rcd1*	Caf40	CAF40 = CNOT9 = Rcd1 = RQCD1
NOT10	CG18616; *Not10*	–	CNOT10
NOT11 = C2orf29	CG13567; *Not11*	–	CNOT11

The CCR4–NOT complex deadenylates mRNAs by means of two exonucleolytic subunits, POP2 and CCR4. *In vitro* assays have shown that orthologs of both proteins from various organisms possess poly(A)-specific 3′ exonuclease activity (Harnisch et al., 2012; Wahle and Winkler, 2013). Such evidence currently does not exist for the *Drosophila* proteins. However, in support of enzymatic activity, overexpression of POP2 carrying point mutations in its active site has dominant negative effects, as discussed below (Temme et al., 2010; Petit et al., 2012). POP2, like PARN and PAN2, the catalytic subunit of the Pan2/Pan3 complex, is a member of the DEDD nuclease family. The eponymous conserved active site residues, which serve to bind two divalent metal ions to catalyze hydrolysis of the phosphodiester bond, are conserved in the *Drosophila* protein. CCR4 has a conserved C-terminal domain responsible for the catalytic activity. Based on sequence alignments and crystal structures, this domain is a member of the exonuclease-endonuclase-phosphatase (EEP) family of enzymes, which, like the DEDD enzymes, catalyze hydrolysis of phosphate ester bonds by a two-metal-ion mechanism (Harnisch et al., 2012; Wahle and Winkler, 2013). In *Drosophila* CCR4, the catalytic domain in general and the active site residues in particular are conserved (Dupressoir et al., 2001). A phenotype caused by a strong hypomorphic allele of the gene encoding CCR4 is only partially rescued by an active site mutant, supporting catalytic activity of the protein, as described in more detail below (Joly et al., 2013).

Interactions between the subunits have been studied by pull-down assays and similar experiments and, more recently, by X-ray crystallography of partial assemblies of the yeast and human complexes (Bhaskar et al., 2013; Boland et al., 2013; Chen et al., 2014a; Mathys et al., 2014; earlier work reviewed in Harnisch et al., 2012; Wahle and Winkler, 2013). Interaction assays in combination with mutagenesis revealed that all interactions are conserved in the *Drosophila* complex (Bawankar et al., 2013; **Figure 1B**). Briefly, NOT1, a large protein of more than 2500 amino acids in flies, the exact size depending on the splice variant, serves as the central scaffold of the complex. Large fragments of NOT1 have been crystallized; all except the CAF40 binding domain (CN9BD for CAF40/CNOT9 binding domain) form HEAT repeats. The central portion of NOT1 associates with POP2, which in turn associates with a leucine-rich repeat (LRR) domain of CCR4 (Basquin et al., 2012; Petit et al., 2012). The active sites of the two exonucleases are quite distant from each other, and their conformations are not affected by incorporation into the complex. NOT2 and NOT3 form a heterodimer via their C-terminal homologous NOT boxes, and the pair binds tightly to the C-terminal portion of NOT1 (Bhaskar et al., 2013; Boland et al., 2013). Binding of CAF40 to the CN9BD, which forms a three helix bundle, is independent of any of the other subunits (Chen et al., 2014a; Mathys et al., 2014). An N-terminal fragment of NOT1 has recently been shown to associate with a NOT10–NOT11 heterodimer in flies and in man (Bawankar et al., 2013; Mauxion et al., 2013).

When the *Drosophila* CCR4–NOT complex was immunopurified by means of a monoclonal antibody directed against NOT1 and elution with the antigenic peptide, all subunits listed were co-precipitated, although NOT10 was at the limit of detection (Temme et al., 2010). NOT4, which was identified as a subunit of the yeast CCR4–NOT complex (Chen et al., 2001), was not found to be associated, in agreement with results obtained in human cells (reviewed in Harnisch et al., 2012; Wahle and Winkler, 2013). All subunits for which antibodies were available, CCR4, POP2 and NOT1-3, were visibly depleted from the supernatant of the immunoprecipitation, suggesting that at least a large fraction resides in the complex. In support of obligatory complex formation, individual RNAi-mediated depletion of POP2 or NOT1-3 led to strongly reduced levels of the other three subunits, presumably due to destabilization of incomplete complexes (Temme et al., 2010; Boland et al., 2013). Similarly, depletion of POP2 reduced the amount of CCR4, and, conversely, ovaries mutant for CCR4 show decreased levels of POP2 (Temme et al., 2004).

Functional studies support the idea that the polypeptides listed assemble for the purpose of mRNA deadenylation. Individual knock-down of POP2, NOT1, NOT2, or NOT3 in Schneider cells led to an increase in bulk poly(A) tail length and a reduced rate of deadenylation of the unstable *Hsp70* mRNA or a reporter mRNA carrying the *Hsp70* 3′ UTR (Temme et al., 2004, 2010; Bönisch et al., 2007; Boland et al., 2013). Note that, because of the co-depletion of other subunits, these experiments only confirm the involvement of the complex in deadenylation but allow no conclusion regarding individual subunits. Knock-down of NOT4 had no effect, in agreement with its absence from the complex. Depletion of CAF40 and, surprisingly, CCR4, also had no effect on bulk poly(A) or the rate of *Hsp70* mRNA deadenylation in Schneider cells (Temme et al., 2010). Overexpression of an inactive point mutant of POP2 retarded deadenylation of CCR4–NOT substrates (Temme et al., 2010; Petit et al., 2012) but overexpression of mutant CCR4 did not (Temme et al., 2010). These experiments suggest that POP2 carries the main catalytic activity, at least in Schneider cells and for the mRNA examined. However, genetic experiments support a role of CCR4 in deadenylation, as will be discussed below. Tethering of any of the subunits of the CCR4–NOT complex to a reporter RNA was sufficient to destabilize and repress the translation of this RNA; presumably, any subunit, when tethered to the RNA, can recruit the entire complex, including the polypeptides relevant for mRNA destabilization and repression (Bawankar et al., 2013). The potential subunit NOT4 was not tested in these assays. CAF40 was as repressive and destabilizing as most of the other subunits. However, NOT10 and NOT11 were notably less effective, and an N-terminal deletion variant of NOT1 unable to associate with NOT10 and 11 was as potent as the wild-type. Thus, NOT10 and 11 may be dispensable for the function of the complex in post-transcriptional control. This is in agreement with knock-down experiments in mammalian cells, which showed no effect of NOT10 or NOT11 depletion on mRNA deadenylation (Mauxion et al., 2013). Interestingly, in the tethering experiments, variants of the complex that should be unable to associate with the catalytic subunits were still able not only to repress but also to destabilize the reporter message (Bawankar et al., 2013). Thus, it is conceivable that either there are additional, yet unknown, contacts between the catalytic subunits and the others or that the complex has a destabilizing function that is independent of deadenylation, for example by stimulating decapping through association with the DEAD box RNA helicase Me31B (Chen et al., 2014a; Mathys et al., 2014). *Drosophila* Me31B and its orthologs in diverse species have been characterized as translational repressors (Nakamura et al., 2001; Weston and Sommerville, 2006), but the yeast ortholog Dhh1p is known to also enhance decapping (Nissan et al., 2010).

*Drosophila* CCR4 is encoded by the *twin* gene (Morris et al., 2005). Flies homozygous for any of the known *twin* alleles are viable, but show various degrees of female sterility, and embryos derived from *twin* mutant mothers have a reduced viability (Temme et al., 2004; Morris et al., 2005; Zaessinger et al., 2006). The molecular basis of these phenotypes will be discussed below. Adult flies bearing a null allelic combination of *twin* have an increased steady-state bulk poly(A) tail length, and the decay of *Hsp70* mRNA in *twin* mutant first instar larvae is slower than in wild-type (Temme et al., 2004). These results support an involvement of CCR4 in deadenylation *in vivo*. NOT2 is encoded by *Regena (Rga*; Frolov et al., 1998). A homozygous strong *Rga* allele causes lethality at embryonic and larval stages (Frolov et al., 1998; Temme et al., 2004). Bulk poly(A) tails are slightly but detectably longer in the mutant (Temme et al., 2004). Genetic studies of the CCR4–NOT complex will be discussed in more detail in the section dealing with developmental functions of the complex.

## PROTEINS RELATED TO THE CCR4–NOT COMPLEX

Proteins discussed in this section are listed in **Table 2**.

**Table 2:** Other genes/proteins discussed in this review.

Name of polypeptide	Annotation symbol; gene	Yeast ortholog(s)	Human ortholog(s)
3635	CG31759	none	PDE12
Angel	CG12273; *angel*	none	ANGEL1, ANGEL2
Nocturnin	CG31299; *curled*	none	Nocturnin = CCRN4L
TOB	CG9214	none	BTG1-4, TOB1, TOB2
GW182	CG31992;* gawky* (*gw*)	none	TNRC6A, B, C
Pan2	CG8232	Pan2	Pan2
Pan3	CG11486	Pan3	Pan3

Most organisms have, in addition to one or several CCR4 orthologs, three types of CCR4-related proteins, called 3635, Angel and Nocturnin. They all share the catalytic domain but lack the LRR that mediates the association of CCR4 with POP2 and, thus, the incorporation into the CCR4–NOT complex (Dupressoir et al., 2001).

The fly protein 3635 is encoded by the gene CG31759. The mammalian 3635 ortholog is identical with phosphodiesterase 12 (PDE12), which was identified as a mitochondrial deadenylating enzyme in humans (Poulsen et al., 2011; Rorbach et al., 2011). Thus, 3635 is not directly related to the CCR4–NOT complex. In agreement with the mitochondrial function of mammalian PDE12, an N-terminal mitochondrial targeting peptide is predicted for the *Drosophila* ortholog (http://ihg.gsf.de/ihg/mitoprot.html; Claros and Vincens, 1996). Co-immunoprecipitation experiments in Schneider cells revealed no association of 3635 with the subunits of the CCR4–NOT complex, and knock-down of the protein (which is expressed in Schneider cells based on RT-PCR analysis) or overexpression of an active site mutant had no effect on the deadenylation of the *Hsp70* mRNA (Temme et al., 2010). In summary, the current evidence suggests no involvement of 3635 in cytoplasmic mRNA deadenylation. *Drosophila* 3635 is encoded in the first intron of the gene *aret*, which complicates a genetic analysis.

*Drosophila* Angel is encoded by the *angel* gene (CG12273; Kurzik-Dumke and Zengerle, 1996), which is located in the intron of another gene (CG30183) on the opposite DNA strand. The protein has a conserved nuclease active site but, as for 3635, experiments in Schneider cells did not provide evidence for an association with the CCR4–NOT complex or a role in the degradation of the *Hsp70* mRNA (Temme et al., 2010). Mammalian Angel (=Ccr4d) is associated with a distant CAF1/POP2 relative, Caf1z (Wagner et al., 2007; Godwin et al., 2013). [Note that Nousch et al. (2013) have come to the conclusion that Caf1z is more closely related to PARN than to CAF1.] Apparently, Caf1z is not conserved in flies.

*Drosophila* Nocturnin is encoded by *curled* (CG31299; Grönke et al., 2009). Nocturnin proteins in other organisms have been shown to have poly(A) degrading activity *in vitro* (Harnisch et al., 2012; Godwin et al., 2013), and active site residues are conserved in the fly protein. Immunoprecipitation experiments suggested that Nocturnin can associate with other subunits of the CCR4–NOT complex in Schneider cells, and expression of an active site mutant delayed deadenylation of the *Hsp70* mRNA (Temme et al., 2010). These data, which reveal a potential involvement in mRNA decay of Nocturnin via the CCR4–NOT complex, are surprising, as Nocturnin is lacking the LRR, which is believed to be indispensable for the association of CCR4 with the complex; thus, these results need to be confirmed by additional experiments. Nocturnin is a cytoplasmic protein in *Drosophila* larvae (Grönke et al., 2009). Homozygous *curled* mutants are viable and fertile; curled wings are the only overt phenotype. Vertebrate Nocturnin is expressed in a circadian rhythm, but the protein is not essential for circadian rhythms, as homozygous knock-out mice do not have a circadian phenotype (Stubblefield et al., 2012). Mouse Nocturnin is also involved in the regulation of several metabolic processes (Stubblefield et al., 2012). In *Drosophila*, expression of Nocturnin is induced by food deprivation, consistent with a role in the regulation of metabolism (Grönke et al., 2009). Beyond that, no connections to metabolism or circadian rhythms have been reported, and mRNA targets of Nocturnin are not known.

## THE *Drosophila* CCR4–NOT COMPLEX IS REQUIRED FOR VIABILITY

In yeast, deadenylation by the CCR4–NOT complex is not essential for viability (Tucker et al., 2001). *Drosophila* mutants have been analyzed for NOT2 (*Rga*; Frolov et al., 1998), NOT3 (Neely et al., 2010) and POP2 (Busseau et al., unpublished data) and are lethal at embryonic to larval stages. In addition, knock-downs of *Not1* and *Pop2* specifically in neuroblasts using RNAi are also lethal (Neumüller et al., 2011). In contrast, a null allelic combination of *twin,* which encodes CCR4, is not lethal but female sterile (Temme et al., 2004; Zaessinger et al., 2006). This requirement of NOT1 and POP2 for viability, and of CCR4 for fertility is conserved in *Caenorhabditis elegans* (Nousch et al., 2013). Because the role of POP2 in deadenylation is more prominent than that of CCR4 in somatic tissues, in both *Drosophila* (Temme et al., 2010) and *C. elegans* (Nousch et al., 2013), these data could be consistent with deadenylation by CCR4–NOT being required for viability, with a major role of POP2 as deadenylase. However, current data cannot rule out that the vital function of the CCR4–NOT complex depends on another of its molecular activities such as translational repression or transcriptional regulation.

A tissue-specific RNAi screen in *Drosophila* has implicated several subunits of CCR4–NOT (NOT1-4) in the function and myofibrillar organization of the heart. The role of NOT3 in heart function was also analyzed in mouse and found to be conserved (Neely et al., 2010). Because treatments with inhibitors of histone deacetylases reduced the impairment of heart function, a role of the CCR4–NOT complex in histone acetylation was proposed to underlie its role in cardiac function. Whether or not CCR4–NOT activity in mRNA deadenylation might be involved was not addressed.

## ROLE OF CCR4–NOT-DEPENDENT DEADENYLATION IN GERM CELLS AND STEM CELLS

Among the subunits of the complex, CCR4 is unique in that *twin* mutants are female sterile, and here data support the notion that impaired deadenylation is responsible for this phenotype. Very tight translational regulation is particularly important in the germline and early embryos, and sterility in *twin* mutant is consistent with CCR4 being absolutely required for this regulation. A recent study showing that a deadenylase-dead form of CCR4 can only partially rescue the *twin* mutant phenotype in germline stem cells indicates that both the deadenylase activity and another role of CCR4 in translational repression are important in these cells (Joly et al., 2013).

In the *Drosophila* female, all germ cells derive from two to three germline stem cells localized at the anterior-most region of the ovary, in a structure called the germarium. The germline stem cells divide asymmetrically to self-renew (generate a new germline stem cell) and produce a cell that differentiates into a cystoblast. The cystoblast then divides four times synchronously to produce a 16-germline-cell cyst, among which 15 cells differentiate as nurse cells and one as the oocyte. *twin* mutants show several defects in oogenesis, namely impaired germline stem cell self-renewal (Joly et al., 2013; see below), defects in the synchronous divisions of the cystoblast leading to either more or less than 16 germ cells per cyst, defects in oocyte specification resulting either in the lack of or two oocytes per cyst, and germ cell death (Morris et al., 2005; Zaessinger et al., 2006). Mitotic defects have been correlated with elongated poly(A) tails of *cyclin A* and *cyclin B* mRNAs and increased levels of the corresponding proteins in the germarium (Morris et al., 2005).

**FIGURE 2:**
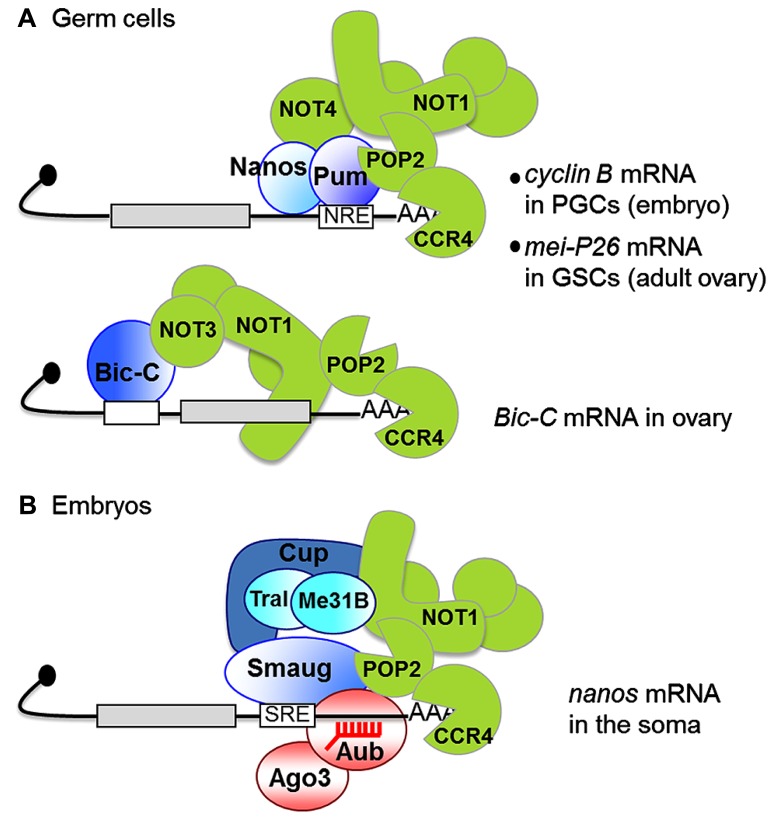
**Interactions between RNA binding proteins and the CCR4–NOT complex in germ cells (A) and early embryos (B)**. Black lines represent mRNAs, gray boxes are coding sequences, white boxes are binding motifs for specific RNA binding proteins [NRE, nanos response element bound by Pumilio (Pum); SRE, Smaug recognition element]. The CCR4–NOT complex is in green. CCR4 is shown degrading the poly(A) tail, but the division of labor between POP2 and CCR4 remains to be analyzed. **(A)** PGCs, primordial germ cells; GSCs, germline stem cells. In contrast to most other examples, the Bicaudal-C (Bic-C) binding element is localized in the 5′ UTR in *Bic-C* mRNA (Chicoine et al., 2007). **(B)** The red comb represent piRNAs. The interaction between Smaug and POP2 is suggested from co-immunoprecipitation assays, but has not been verified *in vitro*. Me31B and Trailer Hitch (Tral) are translational repressors interacting with Cup, which are found in both SRE binding complexes (Jeske et al., 2011) and NOT1 interacting proteins (Temme et al., 2010) in embryo extracts. A direct interaction between the human homolog of Me31B and NOT1 has recently been reported (Chen et al. 2014a; Mathys et al. 2014). A role of Me31B and Tral in Smaug-dependent deadenylation has not been investigated.

The specific requirement for CCR4 in germ cells and early embryos results from the regulation of specific mRNAs by the CCR4–NOT complex in those cells. This is achieved by the recruitment of the complex by mRNA binding proteins. The RNA binding proteins involved in CCR4–NOT-dependent regulation of *cyclin A* and *cyclin B* mRNAs in the germarium have not been identified. However, to date, three RNA binding proteins that interact with the CCR4–NOT complex have been reported in germ cells: Nanos, Pumilio, and Bicaudal-C (**Figure 2A**). Nanos and Pumilio were first shown to mediate *cyclin B* mRNA repression by CCR4–NOT in primordial germ cells, the progenitors of germline stem cells in the embryo (Kadyrova et al., 2007). This regulation is required to limit the proliferation of primordial germ cells before they have migrated to the gonad. Pumilio recognizes specific motifs in the *cyclin B* 3′ UTR and recruits the CCR4–NOT complex through direct interaction with POP2. This Pumilio-POP2 interaction is conserved from yeast to man (Goldstrohm et al., 2006, 2007; Kadyrova et al., 2007; Goldstrohm and Wickens, 2008). Regulation of *cyclin B* also requires Nanos, and, in tethering assays, Nanos alone was able to repress *cyclin B* mRNA. Nanos was found to interact with NOT4 in yeast two-hybrid assays, which might indicate that NOT4 can be a part of the CCR4–NOT complex after all, perhaps in specific tissues. This interaction was not tested *in vivo*, however (Kadyrova et al., 2007). Nanos function in germ cell development and its interaction with the CCR4–NOT complex are conserved in the mouse, where Nanos2 directly binds NOT1 (Suzuki et al., 2010, 2012). This Nanos-NOT1 interaction was recently validated with human proteins by an X-ray structure revealing the association of a short motif in Nanos1 with a hydrophobic pocket in the C-terminal domain of NOT1. This motif is not conserved in *Drosophila* Nanos though, consistent with the recruitment of the CCR4–NOT complex through different interactions (Bhandari et al., 2014).

A role of CCR4 has recently been established in germline stem cells in the adult ovary (Joly et al., 2013). The physical interaction between Nanos/Pumilio and the CCR4–NOT complex has been confirmed in these cells as well, by means of co-immunoprecipitation experiments. Nanos and Pumilio, together with CCR4, have a crucial role in the self-renewal of germline stem cells. This function is thought to result from the translational repression of mRNAs encoding differentiation factors. One of these mRNAs, *mei-P26*, encodes a protein of the Trim-NHL family, which has conserved functions in stem cell biology through the modulation of microRNA (miRNA)-dependent silencing. The CCR4–NOT complex is recruited to *mei-P26* mRNA via binding of Pumilio to its 3′UTR (**Figure 2A**), and the repression of *mei-P26* by CCR4 plays a major role in germline stem cell maintenance, since the loss of germline stem cells in the *twin* mutant is partially rescued by lowering *mei-P26* gene dosage. The fact that the loss of germline stem cells in *twin* mutant ovaries is not completely rescued by a deadenylase-dead form of CCR4 indicates that the deadenylase activity of CCR4 is involved in *twin* function in germline stem cells (Joly et al., 2013). Knock-down of *Not1* in germ cells results in a complete loss of germline stem cells and germ cells in adult ovaries, consistent with the whole CCR4–NOT complex being required for germline stem cell self-renewal (Joly et al., 2013).

The miRNA pathway is also essential for germline stem cell self-renewal in the *Drosophila* ovary (Jin and Xie, 2007; Park et al., 2007; Yang et al., 2007). Although a direct link between components of this pathway and the CCR4–NOT complex is likely for this function (see below), the question has not been addressed yet.

Intriguingly, a role of CCR4–NOT-dependent deadenylation in adult stem cell biology has also recently been described in planarians (Solana et al., 2013). In this species, however, deadenylation, as assayed by knock-down of *Not1*, is required for stem cell differentiation and for the down-regulation of self-renewal mRNAs, in contrast to the situation in *Drosophila*, where deadenylation by CCR4–NOT is required for germline stem cell self-renewal. Because the specificity of the CCR4–NOT complex for mRNAs depends on RNA binding proteins, its potential role in both stem cell self-renewal and differentiation through interaction with different RNA binding proteins is not unexpected. Alternatively, this difference in the requirement of CCR4–NOT for *Drosophila* and planarian stem cell biology might reflect species specificities.

Bicaudal-C (Bic-C) is the third RNA binding protein known to regulate CCR4–NOT function in *Drosophila* oogenesis (**Figure 2A**). Bic-C binds mRNAs encoding proteins involved in oogenesis and cytoskeletal regulation. It directly interacts with the NOT3 subunit of the CCR4–NOT complex and mediates deadenylation of several of these mRNAs, including its own, during the first half of oogenesis (Chicoine et al., 2007).

## ROLE OF THE CCR4–NOT COMPLEX IN EMBRYONIC DEVELOPMENT

Deadenylation by CCR4–NOT also plays a crucial role in early embryonic development. During the two first hours of *Drosophila* embryogenesis, developmental processes depend on maternal mRNAs, after which the zygotic genome takes over and maternal mRNAs are degraded. Females bearing hypomorphic mutant combinations of *twin* produce embryos that die before larval stage and show asynchrony of mitoses in the syncytial embryo, consistent with defective regulation of mRNAs involved in cell cycle control (Zaessinger et al., 2006). In the embryo, a master regulator of maternal mRNA decay at the maternal-to-zygotic transition is the RNA binding protein Smaug (Tadros et al., 2007; Chen et al., 2014b). A mechanistic analysis of Smaug-dependent decay has been performed for two maternal mRNAs, *nanos* and *Hsp83*, and showed that Smaug induces deadenylation by CCR4–NOT (Semotok et al., 2005; Jeske et al., 2006; Zaessinger et al., 2006). Smaug physically interacts with the CCR4–NOT complex, and together these data have led to the idea that the general maternal mRNA decay induced by Smaug in the early embryo depends on its role in the recruitment of CCR4–NOT to mRNAs containing Smaug recognition elements (SREs; **Figure 2B**). SRE-dependent deadenylation observed in a cell-free system derived from early *Drosophila* embryos was ATP-dependent, but the role of ATP in deadenylation has not yet been elucidated (Jeske et al., 2006). Smaug also associates with the protein Cup in the embryo (Nelson et al., 2004). Cup has recently been reported to interact with the CCR4–NOT complex and mediate deadenylation (Igreja and Izaurralde, 2011). Therefore, Cup could participate in the Smaug-dependent tethering of CCR4–NOT to these specific maternal mRNAs.

*nanos* mRNA deadenylation and translational repression by Smaug and the CCR4–NOT complex in the somatic part of the embryo (Zaessinger et al., 2006; Jeske et al., 2011) plays a key role in embryonic patterning, Nanos itself being a translational repressor which represses anterior determinant mRNAs if ectopically expressed anteriorly (Gavis and Lehmann, 1992). Although a functional link between the miRNA pathway and the CCR4–NOT complex has not been addressed in embryos, such a link with another family of small non-coding RNAs, the Piwi-interacting RNAs (piRNAs, 23–30 nt), has been reported for the regulation of *nanos*. piRNAs mostly derive from transposable element sequences in *Drosophila*. Two piRNAs were found to target a region in the *nanos* 3′ UTR by complementarity and nucleate a complex containing the Argonaute proteins Aubergine and Argonaute 3, as well as Smaug and CCR4 (**Figure 2B**). These piRNAs, together with the Argonaute proteins, are required for *nanos* mRNA deadenylation and for anterior–posterior patterning of the embryo (Rouget et al., 2010). Like most other piRNAs, those targeting the *nanos* 3′ UTR are produced from transposable elements. This provides a functional link between transposable elements and mRNA regulation, with an essential role in development.

## OTHER SUBSTRATES AND ACTIVATORS OF DEADENYLATION

As alluded to repeatedly, the *Hsp70* mRNA is a well-characterized substrate for deadenylation by the CCR4–NOT complex (Temme et al., 2004, 2010). Transcription of the gene is induced by heat shock and ceases immediately upon the return of cells to normal growth temperature. Decay of the RNA commences under the same circumstances [half-life 15–30 min (Petersen and Lindquist, 1988)], so that the *Hsp70* mRNA can be used for simple transcriptional pulse-chase experiments in the absence of actinomycin D or other transcription inhibitors. The *Hsp70* 3′ UTR is sufficient to induce rapid deadenylation and decay of a reporter mRNA (Bönisch et al., 2007). However, specific destabilizing sequences have so far not been mapped, and the factor inducing deadenylation has not been identified.

Schneider cells are used to study the innate immune response of *Drosophila*. As a response to stimulation by bacterial peptidoglycan, these cells express several antimicrobial peptides. The mRNAs encoding some of these peptides are induced transiently. For example, the *cecropin A1* (*CecA1*) mRNA has a relatively short half-life of 200 min, and a reporter RNA carrying the *CecA1* 3′ UTR and induced independently of peptidoglycan treatment is even more unstable. Deadenylation and decay of these RNAs is blocked by RNAi-mediated depletion of POP2 and other subunits of the CCR4–NOT complex. Interestingly, AU-rich elements (AREs) in the *CecA1* 3′ UTR and the protein TIS11 are also required for rapid deadenylation (Lauwers et al., 2009; Vindry et al., 2012). TIS11 is the *Drosophila* ortholog of mammalian Tristetraprolin (TTP), the best-studied protein destabilizing mRNAs by binding to AREs (Carballo et al., 1998; Brooks and Blackshear, 2013). TTP is known to induce deadenylation by interaction with the NOT1 subunit of the CCR4–NOT complex (Lykke-Andersen and Wagner, 2005; Marchese et al., 2010; Sandler et al., 2011). A short peptide motif at the very C-terminus of TTP has recently been identified that mediates an interaction with HEAT repeats 10–13 of NOT1. This interaction motif is conserved in *Drosophila* TIS11 (Fabian et al., 2013). Thus, the mechanism of ARE-mediated mRNA decay appears to be conserved between *Drosophila* and man.

The family of TOB/BTG proteins, which has six members in humans, is composed of general activators of deadenylation. The mammalian TOB proteins have an antiproliferative activity in tissue culture cells, which depends on a conserved N-terminal domain (APRO or TOB domain) mediating their association with CAF1/POP2 orthologs. Expression of TOB proteins increases the rate of mRNA deadenylation by mechanisms which are not fully understood, but may involve TOB interacting with specific RNA binding proteins and thus recruiting the CCR4–NOT complex (Horiuchi et al., 2009; Mauxion et al., 2009; Doidge et al., 2012; Ezzeddine et al., 2012; Ogami et al., 2014). The single *Drosophila* TOB protein has not been characterized with respect to its effect on the CCR4–NOT complex, but the residues mediating the interaction between mammalian CAF1 and TOB are mostly conserved in both corresponding *Drosophila* proteins. The two mammalian TOB, but not the BTG proteins, carry, in their C-terminal domains, conserved PAM2 (PABP interacting motif 2) motifs that permit an association with the cytoplasmic poly(A) binding protein; these motifs are also present in *Drosophila* TOB.

MicroRNAs repress gene expression both by inhibiting translation and promoting mRNA decay, and accelerated deadenylation can achieve both. Several recent studies have come to the conclusion that deadenylation and destabilization of mRNAs is the primary mode of action of miRNAs (Huntzinger and Izaurralde, 2011; Subtelny et al., 2014). MicroRNAs promote deadenylation by both CCR4–NOT and Pan2/Pan3 and subsequent 5′ decay of mRNAs in animal cells (Behm-Ansmant et al., 2006; Chen et al., 2009; Fabian et al., 2009; Huntzinger and Izaurralde, 2011). The effects of miRNAs are mediated by GW182 (glycine-tryptophan repeat-containing protein of 182 kDa) proteins, which are components of the miRISC (RNA-induced silencing complex) through interaction with the miRNA-associated Argonaute (Ago) proteins. In a *Drosophila in vitro* system, only Ago1 but not Ago2 induced deadenylation (Iwasaki et al., 2009). This is explained by the fact that only Ago1 interacts with GW182 and consistent with the functional distinction between Ago1 acting in the miRNA pathway and Ago2 acting in RNA interference (Rehwinkel et al., 2005; Behm-Ansmant et al., 2006; Iwasaki et al., 2009). Like SRE-dependent deadenylation (see above), miRNA-induced *in vitro* deadenylation was ATP-dependent. Both in mammalian and in *Drosophila* Schneider cells, GW182 interacts directly with the Pan2/Pan3 as well as the CCR4–NOT complex (Braun et al., 2011; Chekulaeva et al., 2011; Fabian et al., 2011; Huntzinger et al., 2012; Christie et al., 2013; Chen et al., 2014a; Mathys et al., 2014). The interactions, which differ in molecular detail between *Drosophila* and human proteins, are mediated by short tryptophan-containing motifs that are spread over the C-terminal effector or silencing domain and, in the case of the *Drosophila* protein, also additional, more N-terminal sequences of GW182. Corresponding binding pockets for the tryptophan residues of GW182 reside in the C-terminal domain of Pan3, in CAF40, and in NOT1. *Drosophila* GW182, like its mammalian orthologs, also interacts with the cytoplasmic poly(A) binding protein, PABPC (Fabian et al., 2009; Zekri et al., 2009; Huntzinger et al., 2010, 2012; Jinek et al., 2010). Whether or not this interaction is important for miRNA-dependent deadenylation and mRNA repression has been controversial (Fabian and Sonenberg, 2012). Data indicate that PABPC binding contributes to the silencing activity of *Drosophila* and human GW182, although the effect on deadenylation was not examined specifically (Huntzinger et al., 2010, 2012). However, in an *in vitro* system derived from *Drosophila* embryos, miRNA-dependent deadenylation and translational repression were independent of PABPC (Fukaya and Tomari, 2011). *Drosophila* GW182 is encoded by *gawky* (*gw*). Although *gw* message is also supplied maternally, *gw* is among an extremely small group of genes transcribed very early in the embryo, before large-scale zygotic genome activation. In agreement with this very early expression, embryos mutant for zygotic *gw* expression show defects as early as nuclear cycle 10, the beginning of the syncytial blastoderm stage (Schneider et al., 2006). While the molecular basis of the *gw* phenotype has not been investigated, it may be related to the role of miRNAs in the degradation of maternal mRNA during early development (Bushati et al., 2008; Thomsen et al., 2010).

Recruitment of the CCR4–NOT complex to specific mRNAs by dedicated factors seems to be the rule. However, the complex itself appears to be able to bind RNA not only in its nuclease active sites, but also by means of the NOT1–NOT2–NOT3 module (Bhaskar et al., 2013); thus the possibility of an inherent substrate selectivity cannot be dismissed.

## RELATIONSHIP OF CCR4–NOT-DEPENDENT DEADENYLATION TO OTHER ASPECTS OF mRNA DECAY

As mentioned above, there are two other widely conserved deadenylases in addition to the CCR4–NOT complex. The homodimeric enzyme PARN does not appear to be involved in bulk mRNA deadenylation, but instead seems to act on a small set of specific substrates, not all of them mRNAs (Berndt et al., 2012; Yoda et al., 2013), and may play a particular role under stress conditions (Harnisch et al., 2012; Godwin et al., 2013; Nousch et al., 2013; Virtanen et al., 2013). PARN is not conserved in *Drosophila*. The Pan2/Pan3 complex (Harnisch et al., 2012; Wahle and Winkler, 2013), in which Pan2 carries the catalytic activity, does act in general mRNA decay, but its specific role is not entirely clear. In *S. cerevisiae*, Pan2/Pan3 plays a secondary role in mRNA decay, the more important part being played by the CCR4–NOT complex. The action of Pan2/Pan3 appears to be more prominent at the earliest stages of deadenylation (Brown and Sachs, 1998; Tucker et al., 2001). Similar observations have been made in mammalian cells (Yamashita et al., 2005; Zheng et al., 2008; Chen et al., 2009). In *C. elegans*, in which a comprehensive genetic comparison of all three types of deadenylases has been performed, mutants affecting Pan2/Pan3 (like those affecting PARN) had much more subtle phenotypes than mutants affecting the CCR4–NOT complex (Nousch et al., 2013). Pan2 and Pan3 are conserved in *Drosophila*. In agreement with what has been observed in other organisms, knock-down of Pan2/Pan3 in Schneider cells had weaker, sometimes much weaker effects than knock-down of the CCR4–NOT complex (Bönisch et al., 2007; Lauwers et al., 2009; Braun et al., 2011), but a distinct order in which the two deadenylases act was not obvious. While these results have to be interpreted with the caveat that knock-down efficiencies may be different, the fact that depletion of the CCR4–NOT complex by itself produces a strong deadenylation defect is persuasive evidence that this complex acts as the main deadenylase in Schneider cells. In most published studies that we are aware of, the Pan2/Pan3 complex was found to act on the same RNAs as the CCR4–NOT complex, but less efficiently. The question why there are two conserved deadenylase complexes remains to be answered.

Subsequent to deadenylation, mRNAs can be degraded either by the 5′ pathway, consisting of cap hydrolysis and degradation by the 5′ exonuclease XRN1, or by the 3′ pathway, exonucleolytic digestion by the exosome (Houseley and Tollervey, 2009). In budding yeast, the 5′ decay pathway is thought to be dominant. In Schneider cells, deadenylated decay intermediates of the *Hsp70* mRNA accumulated very dramatically upon depletion of the decapping enzyme, and the same was true for a reporter mRNA carrying the *Hsp70* 3′ UTR. Similar, although less dramatic, effects were observed for a more stable reporter mRNA and for the *Hsp83* and *myc* mRNAs (Bönisch et al., 2007) as well as for *CecA1* (Vindry et al., 2012). Finally, miRNA targets are stabilized by the depletion of the DCP1/DCP2 decapping complex (Behm-Ansmant et al., 2006). This might be explained not only by deadenylation triggering decapping, but also by CCR4–NOT-dependent recruitment of Me31B, as extrapolated from the functions of its human and yeast orthologs: human DDX6 binds to the MIF4G domain of NOT1 (Chen et al., 2014a; Mathys et al., 2014), and yeast Dhh1p favors cap hydrolysis (Nissan et al., 2010). Consistent with conserved interactions, Me31B was found in a complex with CCR4–NOT in embryo extract (Temme et al., 2010). In summary, 5′ decay may be the predominant mRNA decay pathway in Schneider cells.

## Conflict of Interest Statement

The authors declare that the research was conducted in the absence of any commercial or financial relationships that could be construed as a potential conflict of interest.

## Note added in proof

A link between piRNAs and CAF1-dependent deadenylation has been validated in mouse spermatogenesis: Gou et al., *Cell Res.* 2014, doi: 10.1038/cr.2014.41. Pachytene piRNAs instruct massive mRNA elimination during late spermatogenesis.
